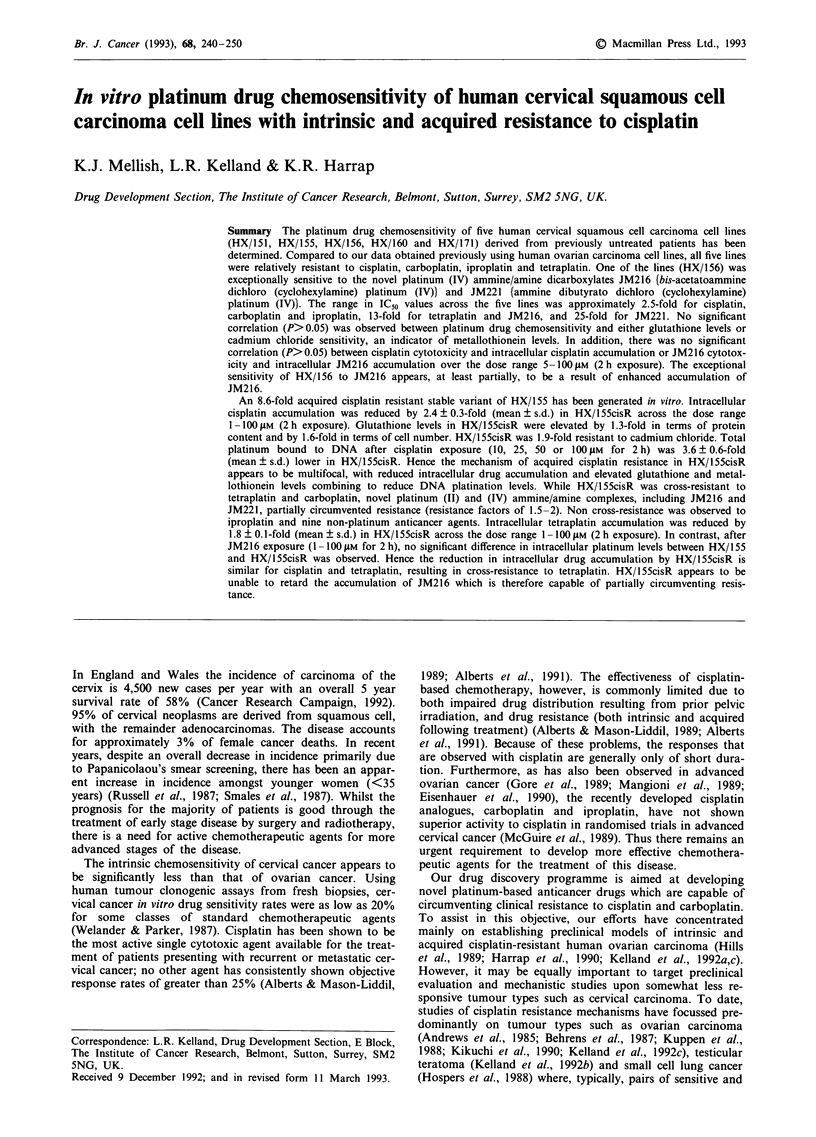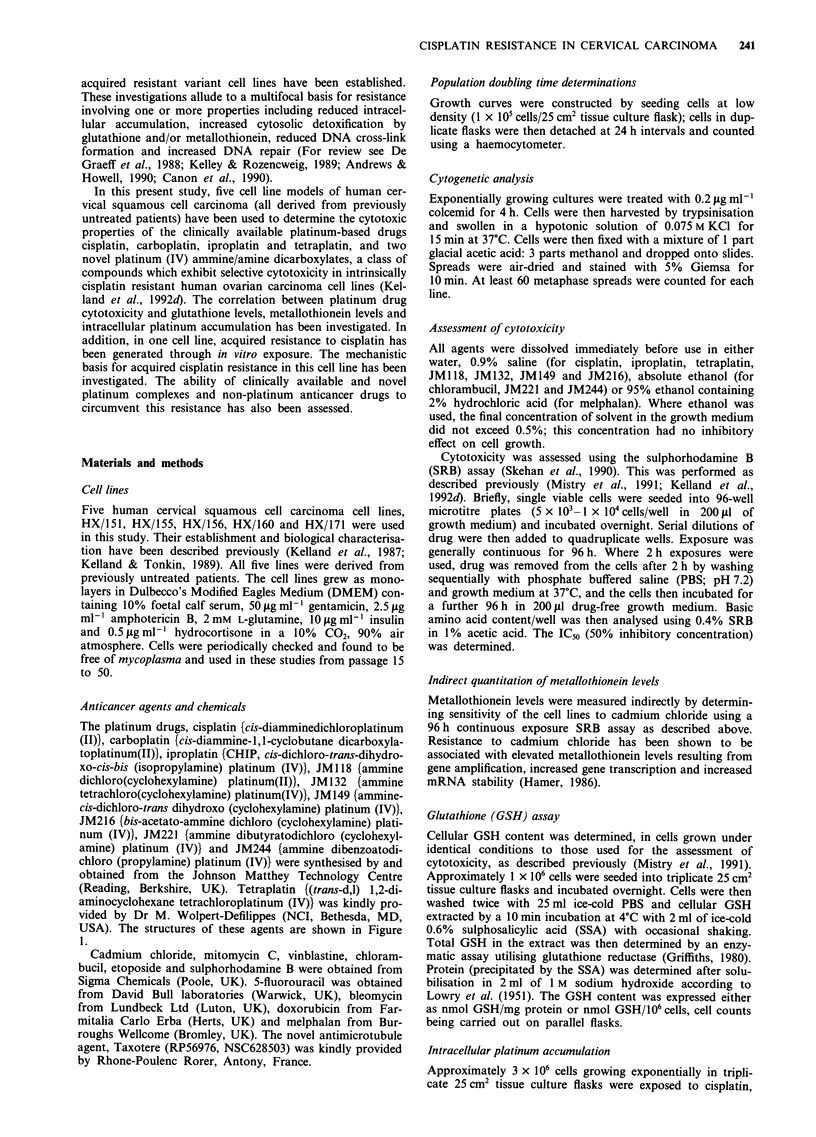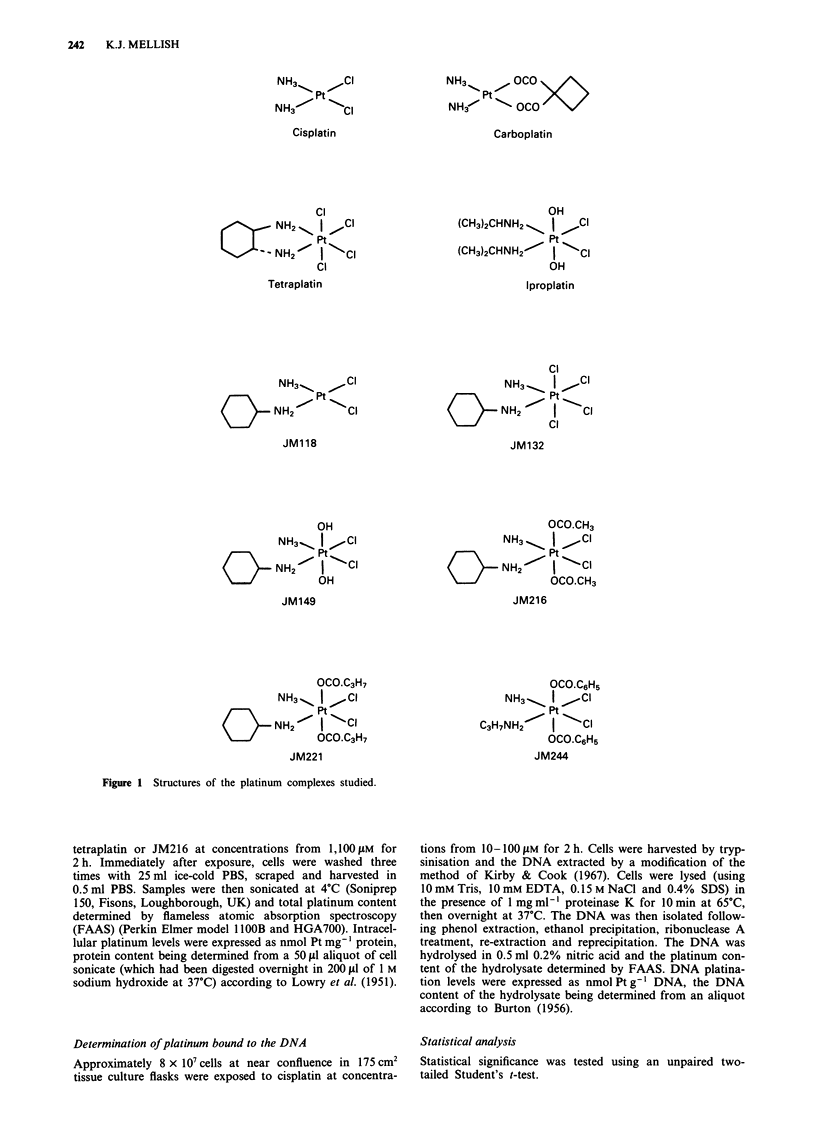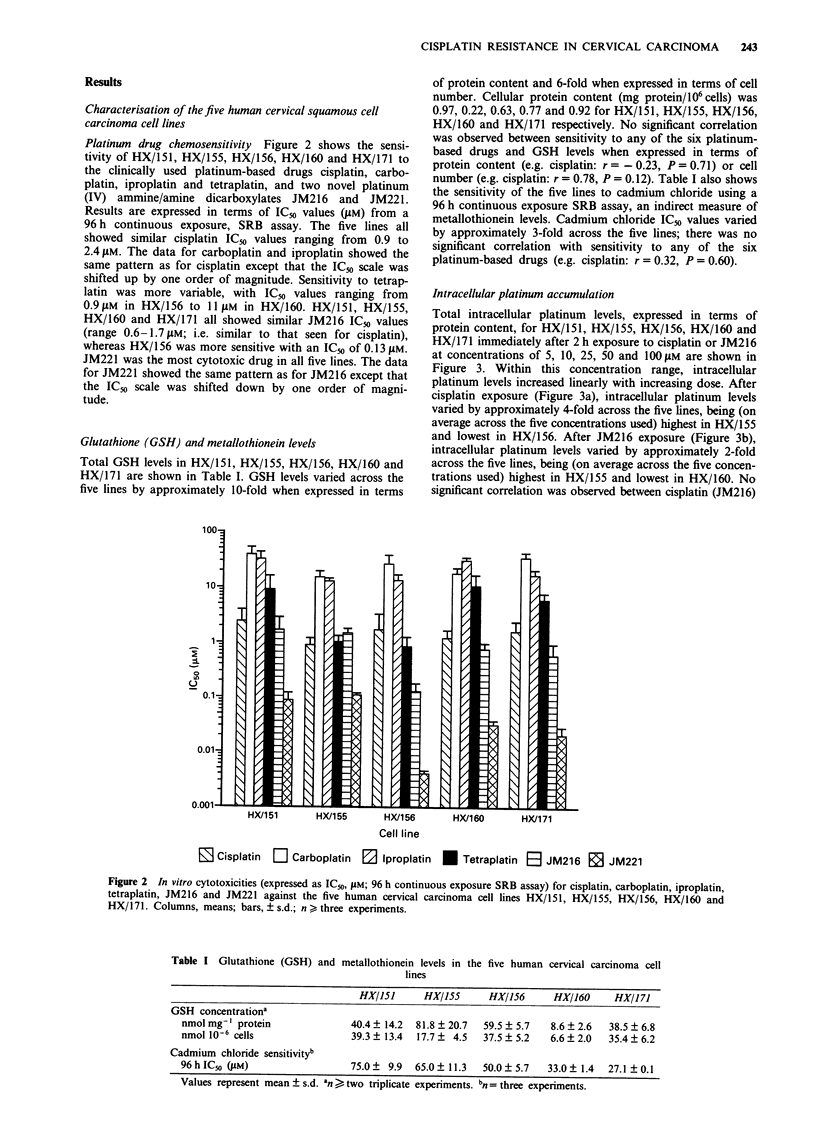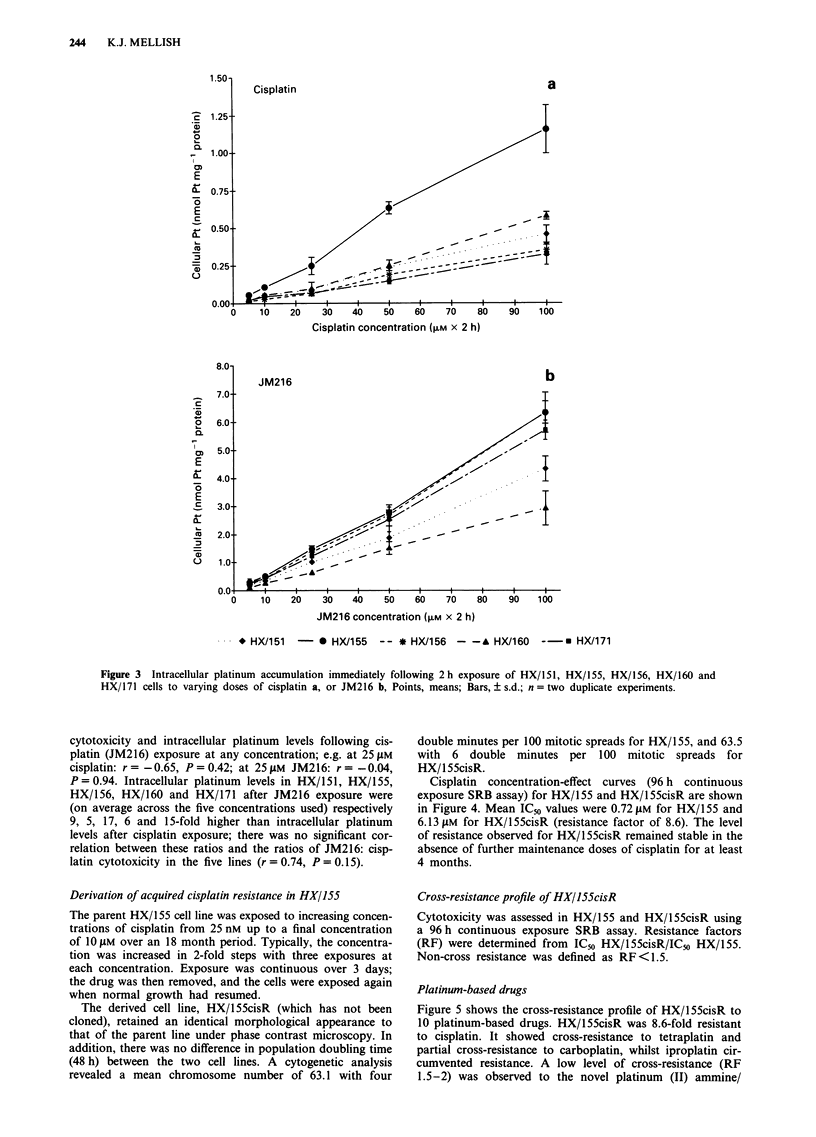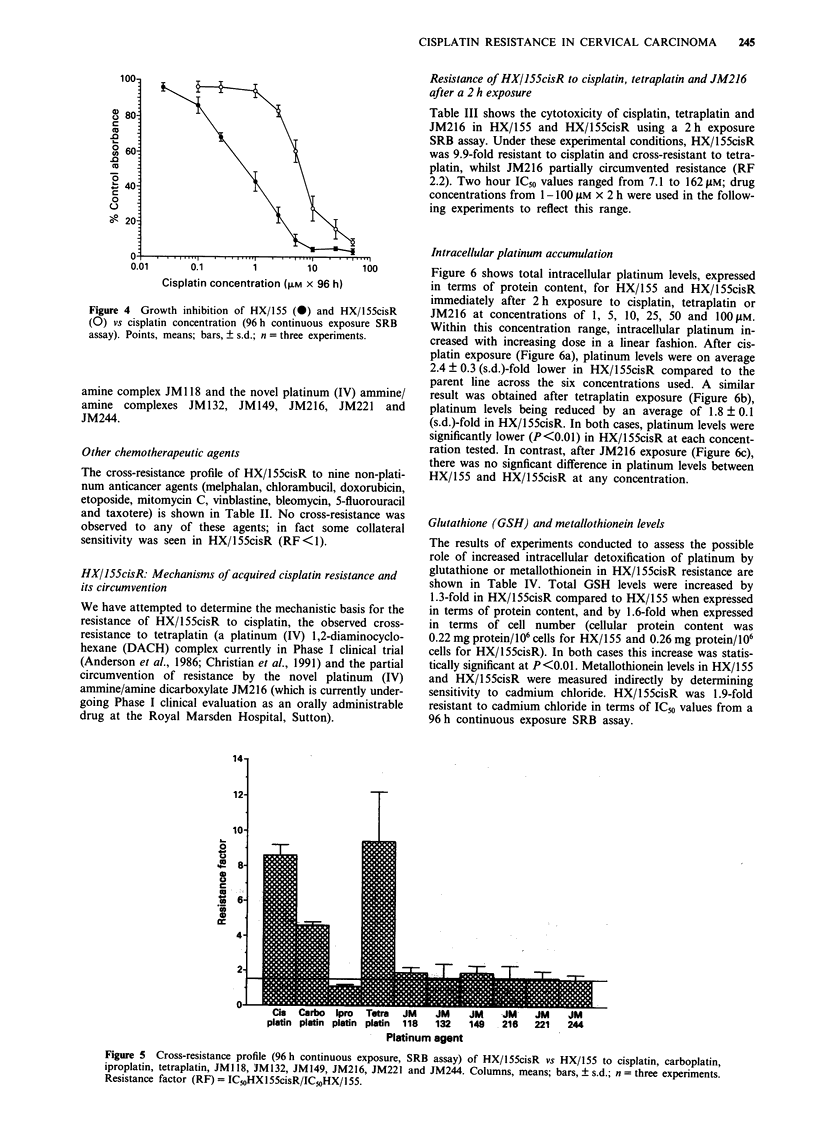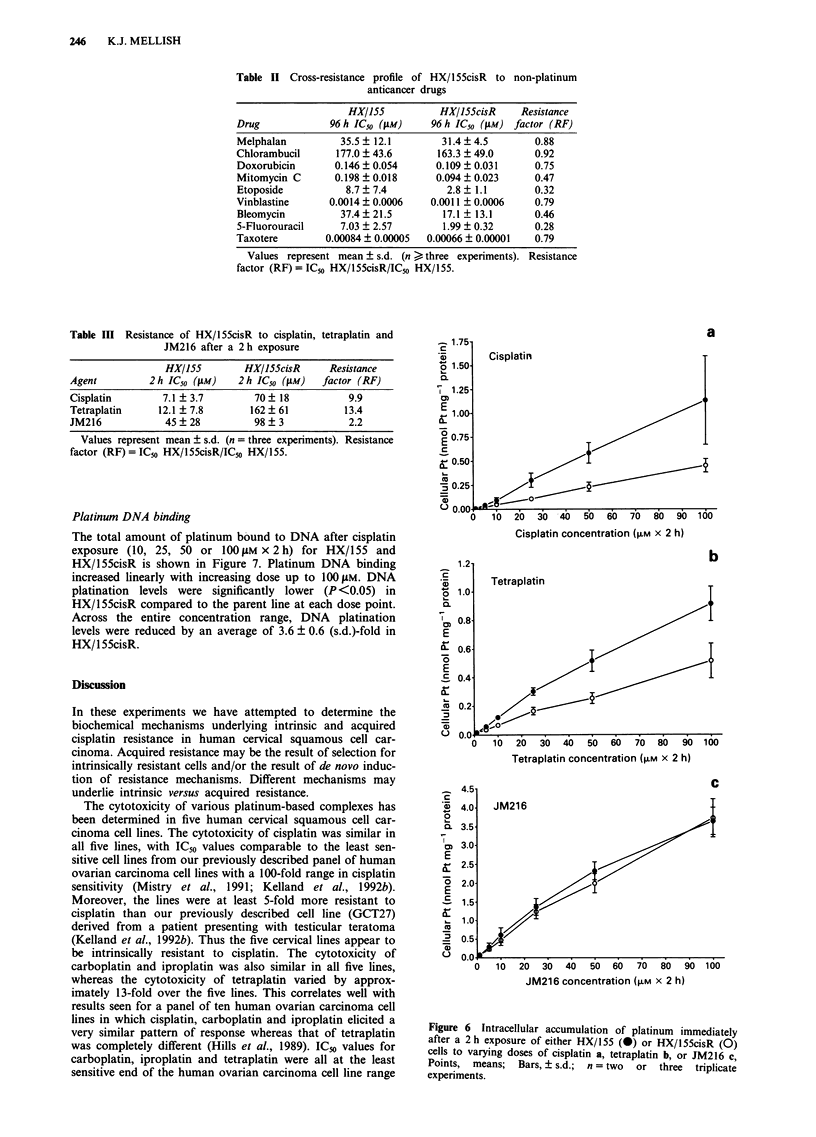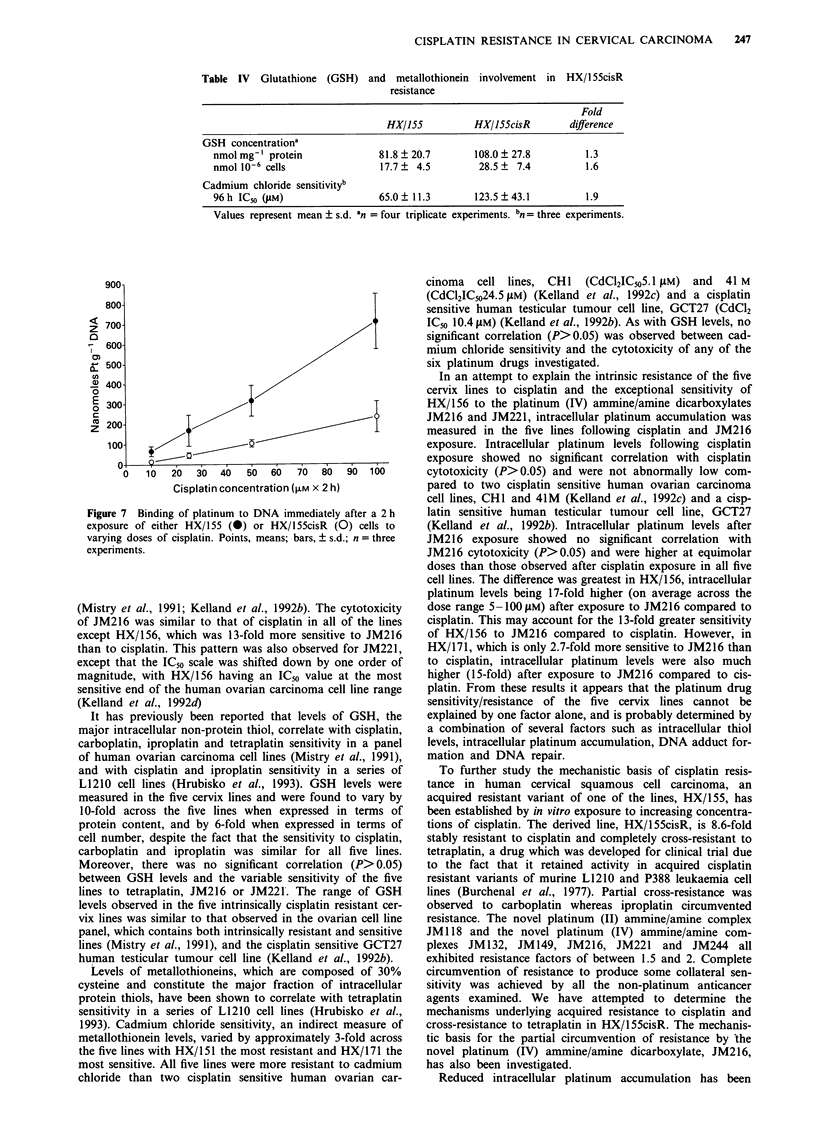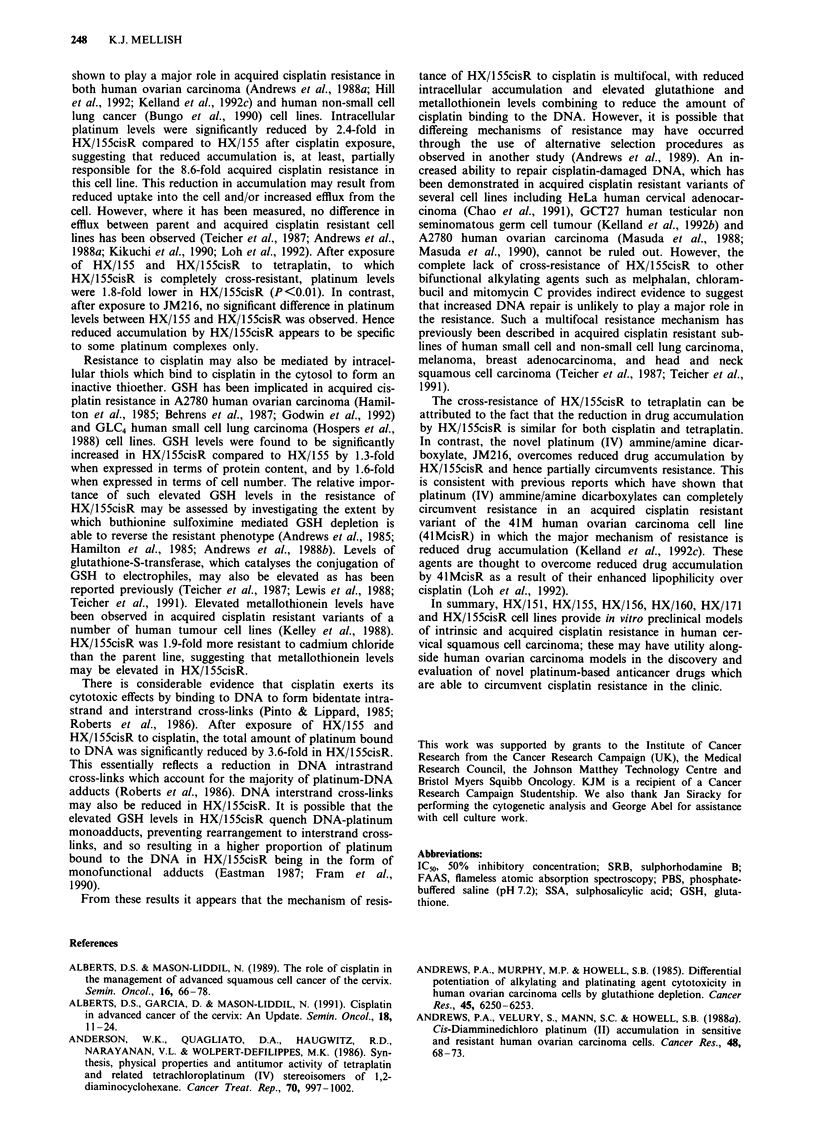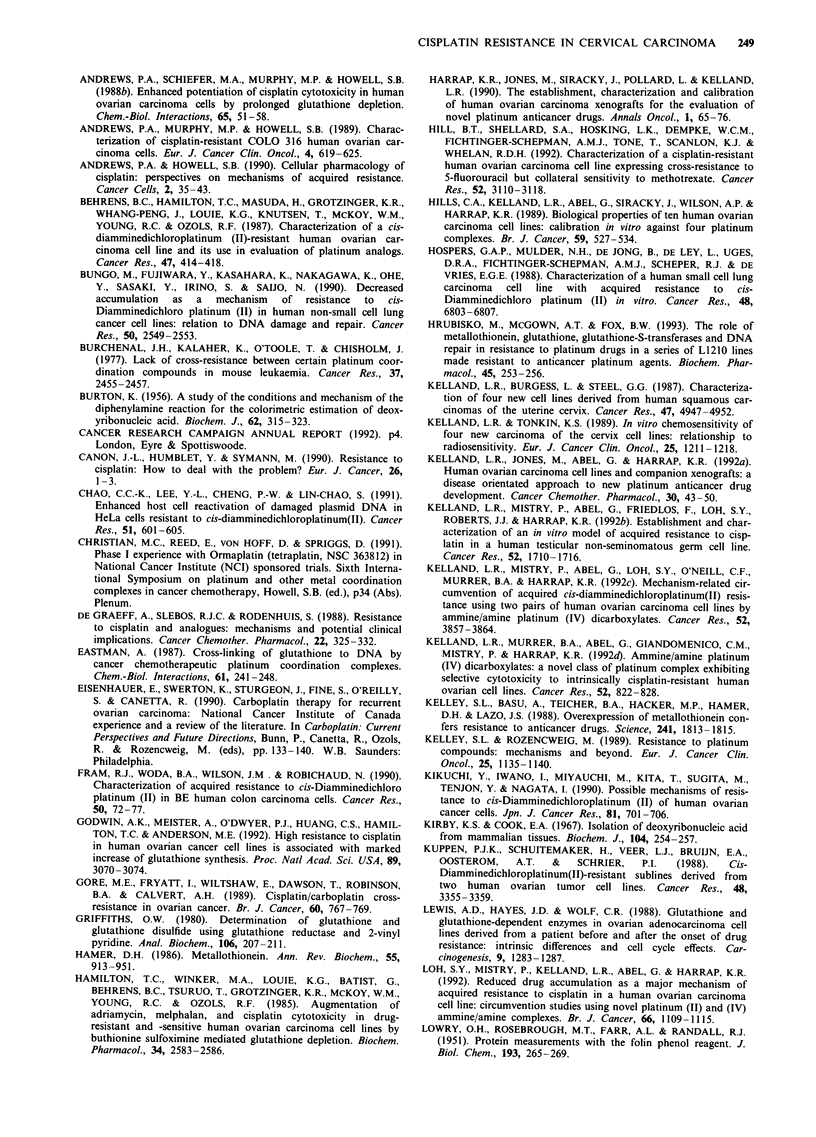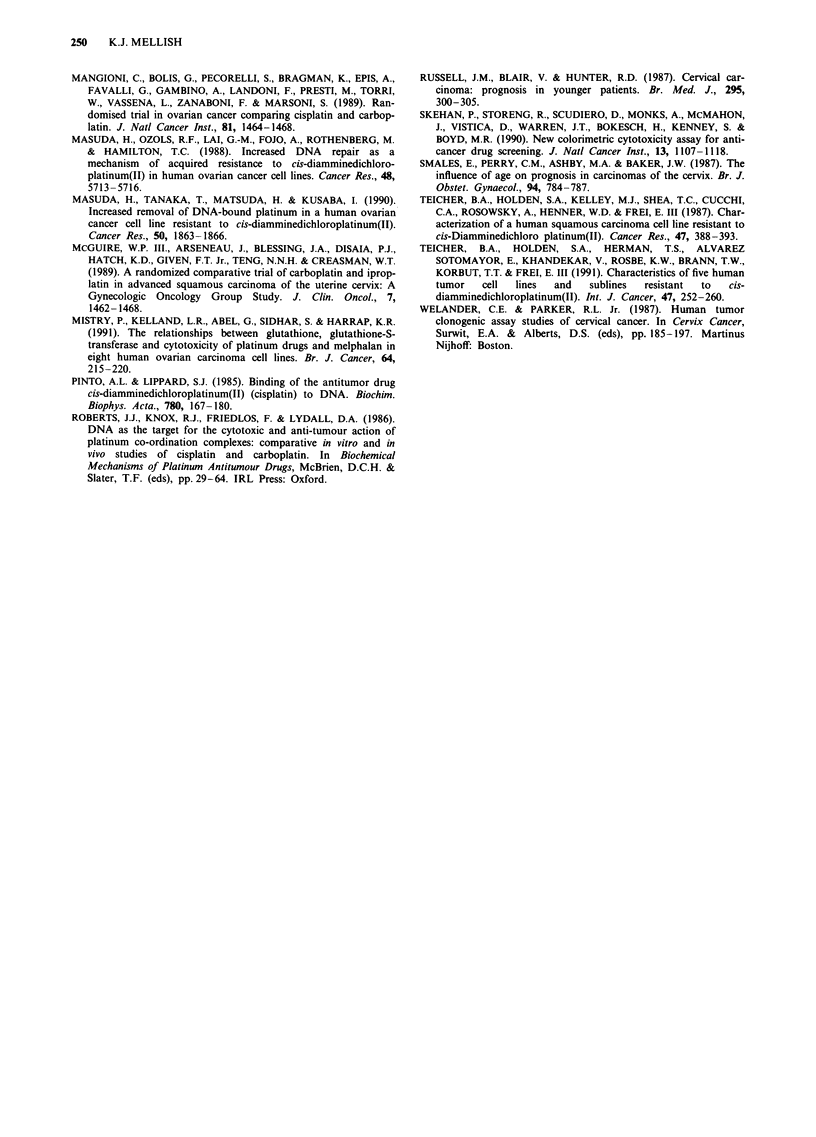# In vitro platinum drug chemosensitivity of human cervical squamous cell carcinoma cell lines with intrinsic and acquired resistance to cisplatin.

**DOI:** 10.1038/bjc.1993.322

**Published:** 1993-08

**Authors:** K. J. Mellish, L. R. Kelland, K. R. Harrap

**Affiliations:** Drug Development Section, Institute of Cancer Research, Sutton, Surrey, UK.

## Abstract

The platinum drug chemosensitivity of five human cervical squamous cell carcinoma cell lines (HX/151, HX/155, HX/156, HX/160 and HX/171) derived from previously untreated patients has been determined. Compared to our data obtained previously using human ovarian carcinoma cell lines, all five lines were relatively resistant to cisplatin, carboplatin, iproplatin and tetraplatin. One of the lines (HX/156) was exceptionally sensitive to the novel platinum (IV) ammine/amine dicarboxylates JM216 [bis-acetatoammine dichloro (cyclohexylamine) platinum (IV)] and JM221 [ammine dibutyrato dichloro (cyclohexylamine) platinum (IV)]. The range in IC50 values across the five lines was approximately 2.5-fold for cisplatin, carboplatin and iproplatin, 13-fold for tetraplatin and JM216, and 25-fold for JM221. No significant correlation (P > 0.05) was observed between platinum drug chemosensitivity and either glutathione levels or cadmium chloride sensitivity, an indicator of metallothionein levels. In addition, there was no significant correlation (P > 0.05) between cisplatin cytotoxicity and intracellular cisplatin accumulation or JM216 cytotoxicity and intracellular JM216 accumulation over the dose range 5-100 microM (2 h exposure). The exceptional sensitivity of HX/156 to JM216 appears, at least partially, to be a result of enhanced accumulation of JM216. An 8.6-fold acquired cisplatin resistant stable variant of HX/155 has been generated in vitro. Intracellular cisplatin accumulation was reduced by 2.4 +/- 0.3-fold (mean +/- s.d.) in HX/155cisR across the dose range 1-100 microM (2 h exposure). Glutathione levels in HX/155cisR were elevated by 1.3-fold in terms of protein content and by 1.6-fold in terms of cell number. HX/155cisR was 1.9-fold resistant to cadmium chloride. Total platinum bound to DNA after cisplatin exposure (10, 25, 50 or 100 microM for 2 h) was 3.6 +/- 0.6-fold (mean +/- s.d.) lower in HX/155cisR. Hence the mechanism of acquired cisplatin resistance in HX/155cisR appears to be multifocal, with reduced intracellular drug accumulation and elevated glutathione and metallothionein levels combining to reduce DNA platination levels. While HX/155cisR was cross-resistant to tetraplatin and carboplatin, novel platinum (II) and (IV) ammine/amine complexes, including JM216 and JM221, partially circumvented resistance (resistance factors of 1.5-2). Non cross-resistance was observed to iproplatin and nine non-platinum anticancer agents. Intracellular tetraplatin accumulation was reduced by 1.8 +/- 0.1-fold (mean +/- s.d.) in HX/155cisR across the dose range 1-100 microM (2 h exposure). In contrast, after JM216 exposure (1-100 microM for 2 h), no significant difference in intracellular platinum levels between HX/155 and HX/155cisR was observed.(ABSTRACT TRUNCATED AT 400 WORDS)


### *In vitro* platinum drug chemosensitivity of human cervical squamous cell carcinoma cell lines with intrinsic and acquired resistance to cisplatin

K.J. Mellish, L.R. Kelland & K.R. Harrap

*Drug Development Section, The Institute of Cancer Research, Belmont, Sutton, Surrey, SM2 5NG, UK.*

**Summary** The platinum drug chemosensitivity of five human cervical squamous cell carcinoma cell lines (HX/151, HX/155, HX/156, HX/160 and HX/171) derived from previously untreated patients has been determined. Compared to our data obtained previously using human ovarian carcinoma cell lines, all five lines were relatively resistant to cisplatin, carboplatin, iproplatin and tetraplatin. One of the lines (HX/156) was exceptionally sensitive to the novel platinum (IV) ammine/amine dicarboxylates JM216 {*bis*-acetatoammine dichloro (cyclohexylamine) platinum (IV)} and JM221 {ammine dibutyrato dichloro (cyclohexylamine) platinum (IV)}. The range in $IC_{50}$ values across the five lines was approximately 2.5-fold for cisplatin, carboplatin and iproplatin, 13-fold for tetraplatin and JM216, and 25-fold for JM221. No significant correlation ($P > 0.05$) was observed between platinum drug chemosensitivity and either glutathione levels or cadmium chloride sensitivity, an indicator of metallothionein levels. In addition, there was no significant correlation ($P > 0.05$) between cisplatin cytotoxicity and intracellular cisplatin accumulation or JM216 cytotoxicity and intracellular JM216 accumulation over the dose range 5–100 μM (2 h exposure). The exceptional sensitivity of HX/156 to JM216 appears, at least partially, to be a result of enhanced accumulation of JM216.

An 8.6-fold acquired cisplatin resistant stable variant of HX/155 has been generated *in vitro*. Intracellular cisplatin accumulation was reduced by 2.4 ± 0.3-fold (mean ± s.d.) in HX/155cisR across the dose range 1–100 μM (2 h exposure). Glutathione levels in HX/155cisR were elevated by 1.3-fold in terms of protein content and by 1.6-fold in terms of cell number. HX/155cisR was 1.9-fold resistant to cadmium chloride. Total platinum bound to DNA after cisplatin exposure (10, 25, 50 or 100 μM for 2 h) was 3.6 ± 0.6-fold (mean ± s.d.) lower in HX/155cisR. Hence the mechanism of acquired cisplatin resistance in HX/155cisR appears to be multifocal, with reduced intracellular drug accumulation and elevated glutathione and metallothionein levels combining to reduce DNA platination levels. While HX/155cisR was cross-resistant to tetraplatin and carboplatin, novel platinum (II) and (IV) ammine/amine complexes, including JM216 and JM221, partially circumvented resistance (resistance factors of 1.5–2). Non cross-resistance was observed to iproplatin and nine non-platinum anticancer agents. Intracellular tetraplatin accumulation was reduced by 1.8 ± 0.1-fold (mean ± s.d.) in HX/155cisR across the dose range 1–100 μM (2 h exposure). In contrast, after JM216 exposure (1–100 μM for 2 h), no significant difference in intracellular platinum levels between HX/155 and HX/155cisR was observed. Hence the reduction in intracellular drug accumulation by HX/155cisR is similar for cisplatin and tetraplatin, resulting in cross-resistance to tetraplatin. HX/155cisR appears to be unable to retard the accumulation of JM216 which is therefore capable of partially circumventing resistance.

In England and Wales the incidence of carcinoma of the cervix is 4,500 new cases per year with an overall 5 year survival rate of 58% (Cancer Research Campaign, 1992). 95% of cervical neoplasms are derived from squamous cell, with the remainder adenocarcinomas. The disease accounts for approximately 3% of female cancer deaths. In recent years, despite an overall decrease in incidence primarily due to Papanicolaou's smear screening, there has been an apparent increase in incidence amongst younger women (<35 years) (Russell *et al.*, 1987; Smales *et al.*, 1987). Whilst the prognosis for the majority of patients is good through the treatment of early stage disease by surgery and radiotherapy, there is a need for active chemotherapeutic agents for more advanced stages of the disease.

The intrinsic chemosensitivity of cervical cancer appears to be significantly less than that of ovarian cancer. Using human tumour clonogenic assays from fresh biopsies, cervical cancer *in vitro* drug sensitivity rates were as low as 20% for some classes of standard chemotherapeutic agents (Welander & Parker, 1987). Cisplatin has been shown to be the most active single cytotoxic agent available for the treatment of patients presenting with recurrent or metastatic cervical cancer; no other agent has consistently shown objective response rates of greater than 25% (Alberts & Mason-Liddil, 1989; Alberts *et al.*, 1991). The effectiveness of cisplatin-based chemotherapy, however, is commonly limited due to both impaired drug distribution resulting from prior pelvic irradiation, and drug resistance (both intrinsic and acquired following treatment) (Alberts & Mason-Liddil, 1989; Alberts *et al.*, 1991). Because of these problems, the responses that are observed with cisplatin are generally only of short duration. Furthermore, as has also been observed in advanced ovarian cancer (Gore *et al.*, 1989; Mangioni *et al.*, 1989; Eisenhauer *et al.*, 1990), the recently developed cisplatin analogues, carboplatin and iproplatin, have not shown superior activity to cisplatin in randomised trials in advanced cervical cancer (McGuire *et al.*, 1989). Thus there remains an urgent requirement to develop more effective chemotherapeutic agents for the treatment of this disease.

Our drug discovery programme is aimed at developing novel platinum-based anticancer drugs which are capable of circumventing clinical resistance to cisplatin and carboplatin. To assist in this objective, our efforts have concentrated mainly on establishing preclinical models of intrinsic and acquired cisplatin-resistant human ovarian carcinoma (Hills *et al.*, 1989; Harrap *et al.*, 1990; Kelland *et al.*, 1992*a,c*). However, it may be equally important to target preclinical evaluation and mechanistic studies upon somewhat less responsive tumour types such as cervical carcinoma. To date, studies of cisplatin resistance mechanisms have focussed predominantly on tumour types such as ovarian carcinoma (Andrews *et al.*, 1985; Behrens *et al.*, 1987; Kuppen *et al.*, 1988; Kikuchi *et al.*, 1990; Kelland *et al.*, 1992*c*), testicular teratoma (Kelland *et al.*, 1992*b*) and small cell lung cancer (Hospers *et al.*, 1988) where, typically, pairs of sensitive and

Correspondence: L.R. Kelland, Drug Development Section, E Block, The Institute of Cancer Research, Belmont, Sutton, Surrey, SM2 5NG, UK.
Received 9 December 1992; and in revised form 11 March 1993.

acquired resistant variant cell lines have been established. These investigations allude to a multifocal basis for resistance involving one or more properties including reduced intracellular accumulation, increased cytosolic detoxification by glutathione and/or metallothionein, reduced DNA cross-link formation and increased DNA repair (For review see De Graeff *et al.*, 1988; Kelley & Rozencweig, 1989; Andrews & Howell, 1990; Canon *et al.*, 1990).

In this present study, five cell line models of human cervical squamous cell carcinoma (all derived from previously untreated patients) have been used to determine the cytotoxic properties of the clinically available platinum-based drugs cisplatin, carboplatin, iproplatin and tetraplatin, and two novel platinum (IV) ammine/amine dicarboxylates, a class of compounds which exhibit selective cytotoxicity in intrinsically cisplatin resistant human ovarian carcinoma cell lines (Kelland *et al.*, 1992*d*). The correlation between platinum drug cytotoxicity and glutathione levels, metallothionein levels and intracellular platinum accumulation has been investigated. In addition, in one cell line, acquired resistance to cisplatin has been generated through *in vitro* exposure. The mechanistic basis for acquired cisplatin resistance in this cell line has been investigated. The ability of clinically available and novel platinum complexes and non-platinum anticancer drugs to circumvent this resistance has also been assessed.

### Materials and methods

### *Cell lines*

Five human cervical squamous cell carcinoma cell lines, HX/151, HX/155, HX/156, HX/160 and HX/171 were used in this study. Their establishment and biological characterisation have been described previously (Kelland *et al.*, 1987; Kelland & Tonkin, 1989). All five lines were derived from previously untreated patients. The cell lines grew as monolayers in Dulbecco's Modified Eagles Medium (DMEM) containing 10% foetal calf serum, 50 $\mu g\ ml^{-1}$ gentamicin, 2.5 $\mu g\ ml^{-1}$ amphotericin B, 2 mM L-glutamine, 10 $\mu g\ ml^{-1}$ insulin and 0.5 $\mu g\ ml^{-1}$ hydrocortisone in a 10% $CO_2$, 90% air atmosphere. Cells were periodically checked and found to be free of *mycoplasma* and used in these studies from passage 15 to 50.

### *Anticancer agents and chemicals*

The platinum drugs, cisplatin {*cis*-diamminedichloroplatinum (II)}, carboplatin {*cis*-diammine-1,1-cyclobutane dicarboxylatoplatinum(II)}, iproplatin {CHIP, *cis*-dichloro-*trans*-dihydroxo-*cis*-*bis* (isopropylamine) platinum (IV)}, JM118 {ammine dichloro(cyclohexylamine) platinum(II)}, JM132 {ammine tetrachloro(cyclohexylamine) platinum(IV)}, JM149 {ammine-*cis*-dichloro-*trans* dihydroxo (cyclohexylamine) platinum (IV)}, JM216 {*bis*-acetato-ammine dichloro (cyclohexylamine) platinum (IV)}, JM221 {ammine dibutyratodichloro (cyclohexylamine) platinum (IV)} and JM244 {ammine dibenzoatodichloro (propylamine) platinum (IV)} were synthesised by and obtained from the Johnson Matthey Technology Centre (Reading, Berkshire, UK). Tetraplatin {(*trans*-d,l) 1,2-diaminocyclohexane tetrachloroplatinum (IV)} was kindly provided by Dr M. Wolpert-Defilippes (NCI, Bethesda, MD, USA). The structures of these agents are shown in Figure 1.

Cadmium chloride, mitomycin C, vinblastine, chlorambucil, etoposide and sulphorhodamine B were obtained from Sigma Chemicals (Poole, UK). 5-fluorouracil was obtained from David Bull laboratories (Warwick, UK), bleomycin from Lundbeck Ltd (Luton, UK), doxorubicin from Farmitalia Carlo Erba (Herts, UK) and melphalan from Burroughs Wellcome (Bromley, UK). The novel antimicrotubule agent, Taxotere (RP56976, NSC628503) was kindly provided by Rhone-Poulenc Rorer, Antony, France.

### *Population doubling time determinations*

Growth curves were constructed by seeding cells at low density ($1 \times 10^5$ cells/25 $cm^2$ tissue culture flask); cells in duplicate flasks were then detached at 24 h intervals and counted using a haemocytometer.

### *Cytogenetic analysis*

Exponentially growing cultures were treated with 0.2 $\mu g\ ml^{-1}$ colcemid for 4 h. Cells were then harvested by trypsinisation and swollen in a hypotonic solution of 0.075 M KCl for 15 min at 37°C. Cells were then fixed with a mixture of 1 part glacial acetic acid: 3 parts methanol and dropped onto slides. Spreads were air-dried and stained with 5% Giemsa for 10 min. At least 60 metaphase spreads were counted for each line.

### *Assessment of cytotoxicity*

All agents were dissolved immediately before use in either water, 0.9% saline (for cisplatin, iproplatin, tetraplatin, JM118, JM132, JM149 and JM216), absolute ethanol (for chlorambucil, JM221 and JM244) or 95% ethanol containing 2% hydrochloric acid (for melphalan). Where ethanol was used, the final concentration of solvent in the growth medium did not exceed 0.5%; this concentration had no inhibitory effect on cell growth.

Cytotoxicity was assessed using the sulphorhodamine B (SRB) assay (Skehan *et al.*, 1990). This was performed as described previously (Mistry *et al.*, 1991; Kelland *et al.*, 1992*d*). Briefly, single viable cells were seeded into 96-well microtitre plates ($5 \times 10^3 - 1 \times 10^4$ cells/well in 200 $\mu l$ of growth medium) and incubated overnight. Serial dilutions of drug were then added to quadruplicate wells. Exposure was generally continuous for 96 h. Where 2 h exposures were used, drug was removed from the cells after 2 h by washing sequentially with phosphate buffered saline (PBS; pH 7.2) and growth medium at 37°C, and the cells then incubated for a further 96 h in 200 $\mu l$ drug-free growth medium. Basic amino acid content/well was then analysed using 0.4% SRB in 1% acetic acid. The $IC_{50}$ (50% inhibitory concentration) was determined.

### *Indirect quantitation of metallothionein levels*

Metallothionein levels were measured indirectly by determining sensitivity of the cell lines to cadmium chloride using a 96 h continuous exposure SRB assay as described above. Resistance to cadmium chloride has been shown to be associated with elevated metallothionein levels resulting from gene amplification, increased gene transcription and increased mRNA stability (Hamer, 1986).

### *Glutathione (GSH) assay*

Cellular GSH content was determined, in cells grown under identical conditions to those used for the assessment of cytotoxicity, as described previously (Mistry *et al.*, 1991). Approximately $1 \times 10^6$ cells were seeded into triplicate 25 $cm^2$ tissue culture flasks and incubated overnight. Cells were then washed twice with 25 ml ice-cold PBS and cellular GSH extracted by a 10 min incubation at 4°C with 2 ml of ice-cold 0.6% sulphosalicylic acid (SSA) with occasional shaking. Total GSH in the extract was then determined by an enzymatic assay utilising glutathione reductase (Griffiths, 1980). Protein (precipitated by the SSA) was determined after solubilisation in 2 ml of 1 M sodium hydroxide according to Lowry *et al.* (1951). The GSH content was expressed either as nmol GSH/mg protein or nmol GSH/$10^6$ cells, cell counts being carried out on parallel flasks.

### *Intracellular platinum accumulation*

Approximately $3 \times 10^6$ cells growing exponentially in triplicate 25 $cm^2$ tissue culture flasks were exposed to cisplatin,

**Figure 1** Structures of the platinum complexes studied.

tetraplatin or JM216 at concentrations from 1,100 $\mu$M for 2 h. Immediately after exposure, cells were washed three times with 25 ml ice-cold PBS, scraped and harvested in 0.5 ml PBS. Samples were then sonicated at 4°C (Soniprep 150, Fisons, Loughborough, UK) and total platinum content determined by flameless atomic absorption spectroscopy (FAAS) (Perkin Elmer model 1100B and HGA700). Intracellular platinum levels were expressed as nmol Pt $mg^{-1}$ protein, protein content being determined from a 50 $\mu$l aliquot of cell sonicate (which had been digested overnight in 200 $\mu$l of 1 M sodium hydroxide at 37°C) according to Lowry *et al.* (1951).

*Determination of platinum bound to the DNA*

Approximately $8 \times 10^7$ cells at near confluence in 175 $cm^2$ tissue culture flasks were exposed to cisplatin at concentrations from 10–100 $\mu$M for 2 h. Cells were harvested by trypsinisation and the DNA extracted by a modification of the method of Kirby & Cook (1967). Cells were lysed (using 10 mM Tris, 10 mM EDTA, 0.15 M NaCl and 0.4% SDS) in the presence of 1 mg $ml^{-1}$ proteinase K for 10 min at 65°C, then overnight at 37°C. The DNA was then isolated following phenol extraction, ethanol precipitation, ribonuclease A treatment, re-extraction and reprecipitation. The DNA was hydrolysed in 0.5 ml 0.2% nitric acid and the platinum content of the hydrolysate determined by FAAS. DNA platination levels were expressed as nmol Pt $g^{-1}$ DNA, the DNA content of the hydrolysate being determined from an aliquot according to Burton (1956).

*Statistical analysis*

Statistical significance was tested using an unpaired two-tailed Student's *t*-test.

### Results

### *Characterisation of the five human cervical squamous cell carcinoma cell lines*

*Platinum drug chemosensitivity* Figure 2 shows the sensitivity of HX/151, HX/155, HX/156, HX/160 and HX/171 to the clinically used platinum-based drugs cisplatin, carboplatin, iproplatin and tetraplatin, and two novel platinum (IV) ammine/amine dicarboxylates JM216 and JM221. Results are expressed in terms of $IC_{50}$ values ($\mu$M) from a 96 h continuous exposure, SRB assay. The five lines all showed similar cisplatin $IC_{50}$ values ranging from 0.9 to 2.4 $\mu$M. The data for carboplatin and iproplatin showed the same pattern as for cisplatin except that the $IC_{50}$ scale was shifted up by one order of magnitude. Sensitivity to tetraplatin was more variable, with $IC_{50}$ values ranging from 0.9 $\mu$M in HX/156 to 11 $\mu$M in HX/160. HX/151, HX/155, HX/160 and HX/171 all showed similar JM216 $IC_{50}$ values (range 0.6–1.7 $\mu$M; i.e. similar to that seen for cisplatin), whereas HX/156 was more sensitive with an $IC_{50}$ of 0.13 $\mu$M. JM221 was the most cytotoxic drug in all five lines. The data for JM221 showed the same pattern as for JM216 except that the $IC_{50}$ scale was shifted down by one order of magnitude.

### *Glutathione (GSH) and metallothionein levels*

Total GSH levels in HX/151, HX/155, HX/156, HX/160 and HX/171 are shown in Table I. GSH levels varied across the five lines by approximately 10-fold when expressed in terms of protein content and 6-fold when expressed in terms of cell number. Cellular protein content (mg protein/$10^6$ cells) was 0.97, 0.22, 0.63, 0.77 and 0.92 for HX/151, HX/155, HX/156, HX/160 and HX/171 respectively. No significant correlation was observed between sensitivity to any of the six platinum-based drugs and GSH levels when expressed in terms of protein content (e.g. cisplatin: $r = -0.23$, $P = 0.71$) or cell number (e.g. cisplatin: $r = 0.78$, $P = 0.12$). Table I also shows the sensitivity of the five lines to cadmium chloride using a 96 h continuous exposure SRB assay, an indirect measure of metallothionein levels. Cadmium chloride $IC_{50}$ values varied by approximately 3-fold across the five lines; there was no significant correlation with sensitivity to any of the six platinum-based drugs (e.g. cisplatin: $r = 0.32$, $P = 0.60$).

### *Intracellular platinum accumulation*

Total intracellular platinum levels, expressed in terms of protein content, for HX/151, HX/155, HX/156, HX/160 and HX/171 immediately after 2 h exposure to cisplatin or JM216 at concentrations of 5, 10, 25, 50 and 100 $\mu$M are shown in Figure 3. Within this concentration range, intracellular platinum levels increased linearly with increasing dose. After cisplatin exposure (Figure 3a), intracellular platinum levels varied by approximately 4-fold across the five lines, being (on average across the five concentrations used) highest in HX/155 and lowest in HX/156. After JM216 exposure (Figure 3b), intracellular platinum levels varied by approximately 2-fold across the five lines, being (on average across the five concentrations used) highest in HX/155 and lowest in HX/160. No significant correlation was observed between cisplatin (JM216)

**Figure 2** *In vitro* cytotoxicities (expressed as $IC_{50}$, $\mu$M; 96 h continuous exposure SRB assay) for cisplatin, carboplatin, iproplatin, tetraplatin, JM216 and JM221 against the five human cervical carcinoma cell lines HX/151, HX/155, HX/156, HX/160 and HX/171. Columns, means; bars, ± s.d.; $n \geq$ three experiments.

**Table I** Glutathione (GSH) and metallothionein levels in the five human cervical carcinoma cell lines

| | *HX/151* | *HX/155* | *HX/156* | *HX/160* | *HX/171* |
|---|---|---|---|---|---|
| GSH concentration[a] | | | | | |
| nmol $mg^{-1}$ protein | 40.4 ± 14.2 | 81.8 ± 20.7 | 59.5 ± 5.7 | 8.6 ± 2.6 | 38.5 ± 6.8 |
| nmol $10^{-6}$ cells | 39.3 ± 13.4 | 17.7 ± 4.5 | 37.5 ± 5.2 | 6.6 ± 2.0 | 35.4 ± 6.2 |
| Cadmium chloride sensitivity[b] | | | | | |
| 96 h $IC_{50}$ ($\mu$M) | 75.0 ± 9.9 | 65.0 ± 11.3 | 50.0 ± 5.7 | 33.0 ± 1.4 | 27.1 ± 0.1 |

Values represent mean ± s.d. [a]$n \geq$ two triplicate experiments. [b]$n$ = three experiments.

**Figure 3** Intracellular platinum accumulation immediately following 2 h exposure of HX/151, HX/155, HX/156, HX/160 and HX/171 cells to varying doses of cisplatin **a**, or JM216 **b**, Points, means; Bars, ± s.d.; $n$ = two duplicate experiments.

cytotoxicity and intracellular platinum levels following cisplatin (JM216) exposure at any concentration; e.g. at 25 μM cisplatin: $r = -0.65$, $P = 0.42$; at 25 μM JM216: $r = -0.04$, $P = 0.94$. Intracellular platinum levels in HX/151, HX/155, HX/156, HX/160 and HX/171 after JM216 exposure were (on average across the five concentrations used) respectively 9, 5, 17, 6 and 15-fold higher than intracellular platinum levels after cisplatin exposure; there was no significant correlation between these ratios and the ratios of JM216: cisplatin cytotoxicity in the five lines ($r = 0.74$, $P = 0.15$).

### *Derivation of acquired cisplatin resistance in HX/155*

The parent HX/155 cell line was exposed to increasing concentrations of cisplatin from 25 nM up to a final concentration of 10 μM over an 18 month period. Typically, the concentration was increased in 2-fold steps with three exposures at each concentration. Exposure was continuous over 3 days; the drug was then removed, and the cells were exposed again when normal growth had resumed.

The derived cell line, HX/155cisR (which has not been cloned), retained an identical morphological appearance to that of the parent line under phase contrast microscopy. In addition, there was no difference in population doubling time (48 h) between the two cell lines. A cytogenetic analysis revealed a mean chromosome number of 63.1 with four double minutes per 100 mitotic spreads for HX/155, and 63.5 with 6 double minutes per 100 mitotic spreads for HX/155cisR.

Cisplatin concentration-effect curves (96 h continuous exposure SRB assay) for HX/155 and HX/155cisR are shown in Figure 4. Mean $IC_{50}$ values were 0.72 μM for HX/155 and 6.13 μM for HX/155cisR (resistance factor of 8.6). The level of resistance observed for HX/155cisR remained stable in the absence of further maintenance doses of cisplatin for at least 4 months.

### *Cross-resistance profile of HX/155cisR*

Cytotoxicity was assessed in HX/155 and HX/155cisR using a 96 h continuous exposure SRB assay. Resistance factors (RF) were determined from $IC_{50}$ HX/155cisR/$IC_{50}$ HX/155. Non-cross resistance was defined as RF<1.5.

### *Platinum-based drugs*

Figure 5 shows the cross-resistance profile of HX/155cisR to 10 platinum-based drugs. HX/155cisR was 8.6-fold resistant to cisplatin. It showed cross-resistance to tetraplatin and partial cross-resistance to carboplatin, whilst iproplatin circumvented resistance. A low level of cross-resistance (RF 1.5–2) was observed to the novel platinum (II) ammine/

**Figure 4** Growth inhibition of HX/155 (●) and HX/155cisR (○) *vs* cisplatin concentration (96 h continuous exposure SRB assay). Points, means; bars, ± s.d.; $n$ = three experiments.

amine complex JM118 and the novel platinum (IV) ammine/amine complexes JM132, JM149, JM216, JM221 and JM244.

*Other chemotherapeutic agents*

The cross-resistance profile of HX/155cisR to nine non-platinum anticancer agents (melphalan, chlorambucil, doxorubicin, etoposide, mitomycin C, vinblastine, bleomycin, 5-fluorouracil and taxotere) is shown in Table II. No cross-resistance was observed to any of these agents; in fact some collateral sensitivity was seen in HX/155cisR (RF<1).

*HX/155cisR: Mechanisms of acquired cisplatin resistance and its circumvention*

We have attempted to determine the mechanistic basis for the resistance of HX/155cisR to cisplatin, the observed cross-resistance to tetraplatin (a platinum (IV) 1,2-diaminocyclohexane (DACH) complex currently in Phase I clinical trial (Anderson *et al.*, 1986; Christian *et al.*, 1991) and the partial circumvention of resistance by the novel platinum (IV) ammine/amine dicarboxylate JM216 (which is currently undergoing Phase I clinical evaluation as an orally administrable drug at the Royal Marsden Hospital, Sutton).

*Resistance of HX/155cisR to cisplatin, tetraplatin and JM216 after a 2 h exposure*

Table III shows the cytotoxicity of cisplatin, tetraplatin and JM216 in HX/155 and HX/155cisR using a 2 h exposure SRB assay. Under these experimental conditions, HX/155cisR was 9.9-fold resistant to cisplatin and cross-resistant to tetraplatin, whilst JM216 partially circumvented resistance (RF 2.2). Two hour $IC_{50}$ values ranged from 7.1 to 162 μM; drug concentrations from 1–100 μM × 2 h were used in the following experiments to reflect this range.

*Intracellular platinum accumulation*

Figure 6 shows total intracellular platinum levels, expressed in terms of protein content, for HX/155 and HX/155cisR immediately after 2 h exposure to cisplatin, tetraplatin or JM216 at concentrations of 1, 5, 10, 25, 50 and 100 μM. Within this concentration range, intracellular platinum increased with increasing dose in a linear fashion. After cisplatin exposure (Figure 6a), platinum levels were on average 2.4 ± 0.3 (s.d.)-fold lower in HX/155cisR compared to the parent line across the six concentrations used. A similar result was obtained after tetraplatin exposure (Figure 6b), platinum levels being reduced by an average of 1.8 ± 0.1 (s.d.)-fold in HX/155cisR. In both cases, platinum levels were significantly lower ($P$<0.01) in HX/155cisR at each concentration tested. In contrast, after JM216 exposure (Figure 6c), there was no signficant difference in platinum levels between HX/155 and HX/155cisR at any concentration.

*Glutathione (GSH) and metallothionein levels*

The results of experiments conducted to assess the possible role of increased intracellular detoxification of platinum by glutathione or metallothionein in HX/155cisR resistance are shown in Table IV. Total GSH levels were increased by 1.3-fold in HX/155cisR compared to HX/155 when expressed in terms of protein content, and by 1.6-fold when expressed in terms of cell number (cellular protein content was 0.22 mg protein/$10^6$ cells for HX/155 and 0.26 mg protein/$10^6$ cells for HX/155cisR). In both cases this increase was statistically significant at $P$<0.01. Metallothionein levels in HX/155 and HX/155cisR were measured indirectly by determining sensitivity to cadmium chloride. HX/155cisR was 1.9-fold resistant to cadmium chloride in terms of $IC_{50}$ values from a 96 h continuous exposure SRB assay.

**Figure 5** Cross-resistance profile (96 h continuous exposure, SRB assay) of HX/155cisR *vs* HX/155 to cisplatin, carboplatin, iproplatin, tetraplatin, JM118, JM132, JM149, JM216, JM221 and JM244. Columns, means; bars, ± s.d.; $n$ = three experiments. Resistance factor (RF) = $IC_{50}$HX155cisR/$IC_{50}$HX/155.

**Table II** Cross-resistance profile of HX/155cisR to non-platinum anticancer drugs

| *Drug* | *HX/155* *96 h* $IC_{50}$ *(μM)* | *HX/155cisR* *96 h* $IC_{50}$ *(μM)* | *Resistance factor (RF)* |
|---|---|---|---|
| Melphalan | 35.5 ± 12.1 | 31.4 ± 4.5 | 0.88 |
| Chlorambucil | 177.0 ± 43.6 | 163.3 ± 49.0 | 0.92 |
| Doxorubicin | 0.146 ± 0.054 | 0.109 ± 0.031 | 0.75 |
| Mitomycin C | 0.198 ± 0.018 | 0.094 ± 0.023 | 0.47 |
| Etoposide | 8.7 ± 7.4 | 2.8 ± 1.1 | 0.32 |
| Vinblastine | 0.0014 ± 0.0006 | 0.0011 ± 0.0006 | 0.79 |
| Bleomycin | 37.4 ± 21.5 | 17.1 ± 13.1 | 0.46 |
| 5-Fluorouracil | 7.03 ± 2.57 | 1.99 ± 0.32 | 0.28 |
| Taxotere | 0.00084 ± 0.00005 | 0.00066 ± 0.00001 | 0.79 |

Values represent mean ± s.d. ($n \geqslant$ three experiments). Resistance factor (RF) = $IC_{50}$ HX/155cisR/$IC_{50}$ HX/155.

**Table III** Resistance of HX/155cisR to cisplatin, tetraplatin and JM216 after a 2 h exposure

| *Agent* | *HX/155* *2 h* $IC_{50}$ *(μM)* | *HX/155cisR* *2 h* $IC_{50}$ *(μM)* | *Resistance factor (RF)* |
|---|---|---|---|
| Cisplatin | 7.1 ± 3.7 | 70 ± 18 | 9.9 |
| Tetraplatin | 12.1 ± 7.8 | 162 ± 61 | 13.4 |
| JM216 | 45 ± 28 | 98 ± 3 | 2.2 |

Values represent mean ± s.d. ($n$ = three experiments). Resistance factor (RF) = $IC_{50}$ HX/155cisR/$IC_{50}$ HX/155.

*Platinum DNA binding*

The total amount of platinum bound to DNA after cisplatin exposure (10, 25, 50 or 100 μM × 2 h) for HX/155 and HX/155cisR is shown in Figure 7. Platinum DNA binding increased linearly with increasing dose up to 100 μM. DNA platination levels were significantly lower ($P < 0.05$) in HX/155cisR compared to the parent line at each dose point. Across the entire concentration range, DNA platination levels were reduced by an average of 3.6 ± 0.6 (s.d.)-fold in HX/155cisR.

### Discussion

In these experiments we have attempted to determine the biochemical mechanisms underlying intrinsic and acquired cisplatin resistance in human cervical squamous cell carcinoma. Acquired resistance may be the result of selection for intrinsically resistant cells and/or the result of *de novo* induction of resistance mechanisms. Different mechanisms may underlie intrinsic *versus* acquired resistance.

The cytotoxicity of various platinum-based complexes has been determined in five human cervical squamous cell carcinoma cell lines. The cytotoxicity of cisplatin was similar in all five lines, with $IC_{50}$ values comparable to the least sensitive cell lines from our previously described panel of human ovarian carcinoma cell lines with a 100-fold range in cisplatin sensitivity (Mistry *et al.*, 1991; Kelland *et al.*, 1992*b*). Moreover, the lines were at least 5-fold more resistant to cisplatin than our previously described cell line (GCT27) derived from a patient presenting with testicular teratoma (Kelland *et al.*, 1992*b*). Thus the five cervical lines appear to be intrinsically resistant to cisplatin. The cytotoxicity of carboplatin and iproplatin was also similar in all five lines, whereas the cytotoxicity of tetraplatin varied by approximately 13-fold over the five lines. This correlates well with results seen for a panel of ten human ovarian carcinoma cell lines in which cisplatin, carboplatin and iproplatin elicited a very similar pattern of response whereas that of tetraplatin was completely different (Hills *et al.*, 1989). $IC_{50}$ values for carboplatin, iproplatin and tetraplatin were all at the least sensitive end of the human ovarian carcinoma cell line range

**Figure 6** Intracellular accumulation of platinum immediately after a 2 h exposure of either HX/155 (●) or HX/155cisR (○) cells to varying doses of cisplatin **a**, tetraplatin **b**, or JM216 **c**. Points, means; Bars, ± s.d.; $n$ = two or three triplicate experiments.

**Table IV** Glutathione (GSH) and metallothionein involvement in HX/155cisR resistance

| | *HX/155* | *HX/155cisR* | *Fold difference* |
|---|---|---|---|
| GSH concentration[a] | | | |
| nmol $mg^{-1}$ protein | 81.8 ± 20.7 | 108.0 ± 27.8 | 1.3 |
| nmol $10^{-6}$ cells | 17.7 ± 4.5 | 28.5 ± 7.4 | 1.6 |
| Cadmium chloride sensitivity[b] | | | |
| 96 h $IC_{50}$ (μM) | 65.0 ± 11.3 | 123.5 ± 43.1 | 1.9 |

Values represent mean ± s.d. [a]$n$ = four triplicate experiments. [b]$n$ = three experiments.

**Figure 7** Binding of platinum to DNA immediately after a 2 h exposure of either HX/155 (●) or HX/155cisR (○) cells to varying doses of cisplatin. Points, means; bars, ± s.d.; $n$ = three experiments.

(Mistry *et al.*, 1991; Kelland *et al.*, 1992*b*). The cytotoxicity of JM216 was similar to that of cisplatin in all of the lines except HX/156, which was 13-fold more sensitive to JM216 than to cisplatin. This pattern was also observed for JM221, except that the $IC_{50}$ scale was shifted down by one order of magnitude, with HX/156 having an $IC_{50}$ value at the most sensitive end of the human ovarian carcinoma cell line range (Kelland *et al.*, 1992*d*)

It has previously been reported that levels of GSH, the major intracellular non-protein thiol, correlate with cisplatin, carboplatin, iproplatin and tetraplatin sensitivity in a panel of human ovarian carcinoma cell lines (Mistry *et al.*, 1991), and with cisplatin and iproplatin sensitivity in a series of L1210 cell lines (Hrubisko *et al.*, 1993). GSH levels were measured in the five cervix lines and were found to vary by 10-fold across the five lines when expressed in terms of protein content, and by 6-fold when expressed in terms of cell number, despite the fact that the sensitivity to cisplatin, carboplatin and iproplatin was similar for all five lines. Moreover, there was no significant correlation ($P > 0.05$) between GSH levels and the variable sensitivity of the five lines to tetraplatin, JM216 or JM221. The range of GSH levels observed in the five intrinsically cisplatin resistant cervix lines was similar to that observed in the ovarian cell line panel, which contains both intrinsically resistant and sensitive lines (Mistry *et al.*, 1991), and the cisplatin sensitive GCT27 human testicular tumour cell line (Kelland *et al.*, 1992*b*).

Levels of metallothioneins, which are composed of 30% cysteine and constitute the major fraction of intracellular protein thiols, have been shown to correlate with tetraplatin sensitivity in a series of L1210 cell lines (Hrubisko *et al.*, 1993). Cadmium chloride sensitivity, an indirect measure of metallothionein levels, varied by approximately 3-fold across the five lines with HX/151 the most resistant and HX/171 the most sensitive. All five lines were more resistant to cadmium chloride than two cisplatin sensitive human ovarian carcinoma cell lines, CH1 ($CdCl_2IC_{50}5.1$ μM) and 41 M ($CdCl_2IC_{50}24.5$ μM) (Kelland *et al.*, 1992*c*) and a cisplatin sensitive human testicular tumour cell line, GCT27 ($CdCl_2$ $IC_{50}$ 10.4 μM) (Kelland *et al.*, 1992*b*). As with GSH levels, no significant correlation ($P > 0.05$) was observed between cadmium chloride sensitivity and the cytotoxicity of any of the six platinum drugs investigated.

In an attempt to explain the intrinsic resistance of the five cervix lines to cisplatin and the exceptional sensitivity of HX/156 to the platinum (IV) ammine/amine dicarboxylates JM216 and JM221, intracellular platinum accumulation was measured in the five lines following cisplatin and JM216 exposure. Intracellular platinum levels following cisplatin exposure showed no significant correlation with cisplatin cytotoxicity ($P > 0.05$) and were not abnormally low compared to two cisplatin sensitive human ovarian carcinoma cell lines, CH1 and 41M (Kelland *et al.*, 1992*c*) and a cisplatin sensitive human testicular tumour cell line, GCT27 (Kelland *et al.*, 1992*b*). Intracellular platinum levels after JM216 exposure showed no significant correlation with JM216 cytotoxicity ($P > 0.05$) and were higher at equimolar doses than those observed after cisplatin exposure in all five cell lines. The difference was greatest in HX/156, intracellular platinum levels being 17-fold higher (on average across the dose range 5–100 μM) after exposure to JM216 compared to cisplatin. This may account for the 13-fold greater sensitivity of HX/156 to JM216 compared to cisplatin. However, in HX/171, which is only 2.7-fold more sensitive to JM216 than to cisplatin, intracellular platinum levels were also much higher (15-fold) after exposure to JM216 compared to cisplatin. From these results it appears that the platinum drug sensitivity/resistance of the five cervix lines cannot be explained by one factor alone, and is probably determined by a combination of several factors such as intracellular thiol levels, intracellular platinum accumulation, DNA adduct formation and DNA repair.

To further study the mechanistic basis of cisplatin resistance in human cervical squamous cell carcinoma, an acquired resistant variant of one of the lines, HX/155, has been established by *in vitro* exposure to increasing concentrations of cisplatin. The derived line, HX/155cisR, is 8.6-fold stably resistant to cisplatin and completely cross-resistant to tetraplatin, a drug which was developed for clinical trial due to the fact that it retained activity in acquired cisplatin resistant variants of murine L1210 and P388 leukaemia cell lines (Burchenal *et al.*, 1977). Partial cross-resistance was observed to carboplatin whereas iproplatin circumvented resistance. The novel platinum (II) ammine/amine complex JM118 and the novel platinum (IV) ammine/amine complexes JM132, JM149, JM216, JM221 and JM244 all exhibited resistance factors of between 1.5 and 2. Complete circumvention of resistance to produce some collateral sensitivity was achieved by all the non-platinum anticancer agents examined. We have attempted to determine the mechanisms underlying acquired resistance to cisplatin and cross-resistance to tetraplatin in HX/155cisR. The mechanistic basis for the partial circumvention of resistance by the novel platinum (IV) ammine/amine dicarboxylate, JM216, has also been investigated.

Reduced intracellular platinum accumulation has been shown to play a major role in acquired cisplatin resistance in both human ovarian carcinoma (Andrews *et al.*, 1988*a*; Hill *et al.*, 1992; Kelland *et al.*, 1992*c*) and human non-small cell lung cancer (Bungo *et al.*, 1990) cell lines. Intracellular platinum levels were significantly reduced by 2.4-fold in HX/155cisR compared to HX/155 after cisplatin exposure, suggesting that reduced accumulation is, at least, partially responsible for the 8.6-fold acquired cisplatin resistance in this cell line. This reduction in accumulation may result from reduced uptake into the cell and/or increased efflux from the cell. However, where it has been measured, no difference in efflux between parent and acquired cisplatin resistant cell lines has been observed (Teicher *et al.*, 1987; Andrews *et al.*, 1988*a*; Kikuchi *et al.*, 1990; Loh *et al.*, 1992). After exposure of HX/155 and HX/155cisR to tetraplatin, to which HX/155cisR is completely cross-resistant, platinum levels were 1.8-fold lower in HX/155cisR ($P<0.01$). In contrast, after exposure to JM216, no significant difference in platinum levels between HX/155 and HX/155cisR was observed. Hence reduced accumulation by HX/155cisR appears to be specific to some platinum complexes only.

Resistance to cisplatin may also be mediated by intracellular thiols which bind to cisplatin in the cytosol to form an inactive thioether. GSH has been implicated in acquired cisplatin resistance in A2780 human ovarian carcinoma (Hamilton *et al.*, 1985; Behrens *et al.*, 1987; Godwin *et al.*, 1992) and $GLC_4$ human small cell lung carcinoma (Hospers *et al.*, 1988) cell lines. GSH levels were found to be significantly increased in HX/155cisR compared to HX/155 by 1.3-fold when expressed in terms of protein content, and by 1.6-fold when expressed in terms of cell number. The relative importance of such elevated GSH levels in the resistance of HX/155cisR may be assessed by investigating the extent by which buthionine sulfoximine mediated GSH depletion is able to reverse the resistant phenotype (Andrews *et al.*, 1985; Hamilton *et al.*, 1985; Andrews *et al.*, 1988*b*). Levels of glutathione-S-transferase, which catalyses the conjugation of GSH to electrophiles, may also be elevated as has been reported previously (Teicher *et al.*, 1987; Lewis *et al.*, 1988; Teicher *et al.*, 1991). Elevated metallothionein levels have been observed in acquired cisplatin resistant variants of a number of human tumour cell lines (Kelley *et al.*, 1988). HX/155cisR was 1.9-fold more resistant to cadmium chloride than the parent line, suggesting that metallothionein levels may be elevated in HX/155cisR.

There is considerable evidence that cisplatin exerts its cytotoxic effects by binding to DNA to form bidentate intrastrand and interstrand cross-links (Pinto & Lippard, 1985; Roberts *et al.*, 1986). After exposure of HX/155 and HX/155cisR to cisplatin, the total amount of platinum bound to DNA was significantly reduced by 3.6-fold in HX/155cisR. This essentially reflects a reduction in DNA intrastrand cross-links which account for the majority of platinum-DNA adducts (Roberts *et al.*, 1986). DNA interstrand cross-links may also be reduced in HX/155cisR. It is possible that the elevated GSH levels in HX/155cisR quench DNA-platinum monoadducts, preventing rearrangement to interstrand cross-links, and so resulting in a higher proportion of platinum bound to the DNA in HX/155cisR being in the form of monofunctional adducts (Eastman 1987; Fram *et al.*, 1990).

From these results it appears that the mechanism of resistance of HX/155cisR to cisplatin is multifocal, with reduced intracellular accumulation and elevated glutathione and metallothionein levels combining to reduce the amount of cisplatin binding to the DNA. However, it is possible that differeing mechanisms of resistance may have occurred through the use of alternative selection procedures as observed in another study (Andrews *et al.*, 1989). An increased ability to repair cisplatin-damaged DNA, which has been demonstrated in acquired cisplatin resistant variants of several cell lines including HeLa human cervical adenocarcinoma (Chao *et al.*, 1991), GCT27 human testicular non seminomatous germ cell tumour (Kelland *et al.*, 1992*b*) and A2780 human ovarian carcinoma (Masuda *et al.*, 1988; Masuda *et al.*, 1990), cannot be ruled out. However, the complete lack of cross-resistance of HX/155cisR to other bifunctional alkylating agents such as melphalan, chlorambucil and mitomycin C provides indirect evidence to suggest that increased DNA repair is unlikely to play a major role in the resistance. Such a multifocal resistance mechanism has previously been described in acquired cisplatin resistant sublines of human small cell and non-small cell lung carcinoma, melanoma, breast adenocarcinoma, and head and neck squamous cell carcinoma (Teicher *et al.*, 1987; Teicher *et al.*, 1991).

The cross-resistance of HX/155cisR to tetraplatin can be attributed to the fact that the reduction in drug accumulation by HX/155cisR is similar for both cisplatin and tetraplatin. In contrast, the novel platinum (IV) ammine/amine dicarboxylate, JM216, overcomes reduced drug accumulation by HX/155cisR and hence partially circumvents resistance. This is consistent with previous reports which have shown that platinum (IV) ammine/amine dicarboxylates can completely circumvent resistance in an acquired cisplatin resistant variant of the 41M human ovarian carcinoma cell line (41McisR) in which the major mechanism of resistance is reduced drug accumulation (Kelland *et al.*, 1992*c*). These agents are thought to overcome reduced drug accumulation by 41McisR as a result of their enhanced lipophilicity over cisplatin (Loh *et al.*, 1992).

In summary, HX/151, HX/155, HX/156, HX/160, HX/171 and HX/155cisR cell lines provide *in vitro* preclinical models of intrinsic and acquired cisplatin resistance in human cervical squamous cell carcinoma; these may have utility alongside human ovarian carcinoma models in the discovery and evaluation of novel platinum-based anticancer drugs which are able to circumvent cisplatin resistance in the clinic.

This work was supported by grants to the Institute of Cancer Research from the Cancer Research Campaign (UK), the Medical Research Council, the Johnson Matthey Technology Centre and Bristol Myers Squibb Oncology. KJM is a recipient of a Cancer Research Campaign Studentship. We also thank Jan Siracky for performing the cytogenetic analysis and George Abel for assistance with cell culture work.

**Abbreviations:**
$IC_{50}$, 50% inhibitory concentration; SRB, sulphorhodamine B; FAAS, flameless atomic absorption spectroscopy; PBS, phosphate-buffered saline (pH 7.2); SSA, sulphosalicylic acid; GSH, glutathione.

### References

ALBERTS, D.S. & MASON-LIDDIL, N. (1989). The role of cisplatin in the management of advanced squamous cell cancer of the cervix. *Semin. Oncol.*, **16**, 66–78.

ALBERTS, D.S., GARCIA, D. & MASON-LIDDIL, N. (1991). Cisplatin in advanced cancer of the cervix: An Update. *Semin. Oncol.*, **18**, 11–24.

ANDERSON, W.K., QUAGLIATO, D.A., HAUGWITZ, R.D., NARAYANAN, V.L. & WOLPERT-DEFILIPPES, M.K. (1986). Synthesis, physical properties and antitumor activity of tetraplatin and related tetrachloroplatinum (IV) stereoisomers of 1,2-diaminocyclohexane. *Cancer Treat. Rep.*, **70**, 997–1002.

ANDREWS, P.A., MURPHY, M.P. & HOWELL, S.B. (1985). Differential potentiation of alkylating and platinating agent cytotoxicity in human ovarian carcinoma cells by glutathione depletion. *Cancer Res.*, **45**, 6250–6253.

ANDREWS, P.A., VELURY, S., MANN, S.C. & HOWELL, S.B. (1988*a*). *Cis*-Diamminedichloro platinum (II) accumulation in sensitive and resistant human ovarian carcinoma cells. *Cancer Res.*, **48**, 68–73.

ANDREWS, P.A., SCHIEFER, M.A., MURPHY, M.P. & HOWELL, S.B. (1988*b*). Enhanced potentiation of cisplatin cytotoxicity in human ovarian carcinoma cells by prolonged glutathione depletion. *Chem.-Biol. Interactions*, **65**, 51–58.

ANDREWS, P.A., MURPHY, M.P. & HOWELL, S.B. (1989). Characterization of cisplatin-resistant COLO 316 human ovarian carcinoma cells. *Eur. J. Cancer Clin. Oncol.*, **4**, 619–625.

ANDREWS, P.A. & HOWELL, S.B. (1990). Cellular pharmacology of cisplatin: perspectives on mechanisms of acquired resistance. *Cancer Cells*, **2**, 35–43.

BEHRENS, B.C., HAMILTON, T.C., MASUDA, H., GROTZINGER, K.R., WHANG-PENG, J., LOUIE, K.G., KNUTSEN, T., MCKOY, W.M., YOUNG, R.C. & OZOLS, R.F. (1987). Characterization of a *cis*-diamminedichloroplatinum (II)-resistant human ovarian carcinoma cell line and its use in evaluation of platinum analogs. *Cancer Res.*, **47**, 414–418.

BUNGO, M., FUJIWARA, Y., KASAHARA, K., NAKAGAWA, K., OHE, Y., SASAKI, Y., IRINO, S. & SAIJO, N. (1990). Decreased accumulation as a mechanism of resistance to *cis*-Diamminedichloro platinum (II) in human non-small cell lung cancer cell lines: relation to DNA damage and repair. *Cancer Res.*, **50**, 2549–2553.

BURCHENAL, J.H., KALAHER, K., O'TOOLE, T. & CHISHOLM, J. (1977). Lack of cross-resistance between certain platinum coordination compounds in mouse leukaemia. *Cancer Res.*, **37**, 2455–2457.

BURTON, K. (1956). A study of the conditions and mechanism of the diphenylamine reaction for the colorimetric estimation of deoxyribonucleic acid. *Biochem. J.*, **62**, 315–323.

CANCER RESEARCH CAMPAIGN ANNUAL REPORT (1992). p4. London, Eyre & Spottiswoode.

CANON, J.-L., HUMBLET, Y. & SYMANN, M. (1990). Resistance to cisplatin: How to deal with the problem? *Eur. J. Cancer*, **26**, 1–3.

CHAO, C.C.-K., LEE, Y.-L., CHENG, P.-W. & LIN-CHAO, S. (1991). Enhanced host cell reactivation of damaged plasmid DNA in HeLa cells resistant to *cis*-diamminedichloroplatinum(II). *Cancer Res.*, **51**, 601–605.

CHRISTIAN, M.C., REED, E., VON HOFF, D. & SPRIGGS, D. (1991). Phase I experience with Ormaplatin (tetraplatin, NSC 363812) in National Cancer Institute (NCI) sponsored trials. Sixth International Symposium on platinum and other metal coordination complexes in cancer chemotherapy, Howell, S.B. (ed.), p34 (Abs). Plenum.

DE GRAEFF, A., SLEBOS, R.J.C. & RODENHUIS, S. (1988). Resistance to cisplatin and analogues: mechanisms and potential clinical implications. *Cancer Chemother. Pharmacol.*, **22**, 325–332.

EASTMAN, A. (1987). Cross-linking of glutathione to DNA by cancer chemotherapeutic platinum coordination complexes. *Chem.-Biol. Interactions*, **61**, 241–248.

EISENHAUER, E., SWERTON, K., STURGEON, J., FINE, S., O'REILLY, S. & CANETTA, R. (1990). Carboplatin therapy for recurrent ovarian carcinoma: National Cancer Institute of Canada experience and a review of the literature. In *Carboplatin: Current Perspectives and Future Directions*, Bunn, P., Canetta, R., Ozols, R. & Rozencweig, M. (eds), pp. 133–140. W.B. Saunders: Philadelphia.

FRAM, R.J., WODA, B.A., WILSON, J.M . & ROBICHAUD, N. (1990). Characterization of acquired resistance to *cis*-Diamminedichloro platinum (II) in BE human colon carcinoma cells. *Cancer Res.*, **50**, 72–77.

GODWIN, A.K., MEISTER, A., O'DWYER, P.J., HUANG, C.S., HAMILTON, T.C. & ANDERSON, M.E. (1992). High resistance to cisplatin in human ovarian cancer cell lines is associated with marked increase of glutathione synthesis. *Proc. Natl Acad. Sci. USA*, **89**, 3070–3074.

GORE, M.E., FRYATT, I., WILTSHAW, E., DAWSON, T., ROBINSON, B.A. & CALVERT, A.H. (1989). Cisplatin/carboplatin cross-resistance in ovarian cancer. *Br. J. Cancer*, **60**, 767–769.

GRIFFITHS, O.W. (1980). Determination of glutathione and glutathione disulfide using glutathione reductase and 2-vinyl pyridine. *Anal. Biochem.*, **106**, 207–211.

HAMER, D.H. (1986). Metallothionein. *Ann. Rev. Biochem.*, **55**, 913–951.

HAMILTON, T.C., WINKER, M.A., LOUIE, K.G., BATIST, G., BEHRENS, B.C., TSURUO, T., GROTZINGER, K.R., MCKOY, W.M., YOUNG, R.C. & OZOLS, R.F. (1985). Augmentation of adriamycin, melphalan, and cisplatin cytotoxicity in drug-resistant and -sensitive human ovarian carcinoma cell lines by buthionine sulfoximine mediated glutathione depletion. *Biochem. Pharmacol.*, **34**, 2583–2586.

HARRAP, K.R., JONES, M., SIRACKY, J., POLLARD, L. & KELLAND, L.R. (1990). The establishment, characterization and calibration of human ovarian carcinoma xenografts for the evaluation of novel platinum anticancer drugs. *Annals Oncol.*, **1**, 65–76.

HILL, B.T., SHELLARD, S.A., HOSKING, L.K., DEMPKE, W.C.M., FICHTINGER-SCHEPMAN, A.M.J., TONE, T., SCANLON, K.J. & WHELAN, R.D.H. (1992). Characterization of a cisplatin-resistant human ovarian carcinoma cell line expressing cross-resistance to 5-fluorouracil but collateral sensitivity to methotrexate. *Cancer Res.*, **52**, 3110–3118.

HILLS, C.A., KELLAND, L.R., ABEL, G., SIRACKY, J., WILSON, A.P. & HARRAP, K.R. (1989). Biological properties of ten human ovarian carcinoma cell lines: calibration *in vitro* against four platinum complexes. *Br. J. Cancer*, **59**, 527–534.

HOSPERS, G.A.P., MULDER, N.H., DE JONG, B., DE LEY, L., UGES, D.R.A., FICHTINGER-SCHEPMAN, A.M.J., SCHEPER, R.J. & DE VRIES, E.G.E. (1988). Characterization of a human small cell lung carcinoma cell line with acquired resistance to *cis*-Diamminedichloro platinum (II) *in vitro*. *Cancer Res.*, **48**, 6803–6807.

HRUBISKO, M., MCGOWN, A.T. & FOX, B.W. (1993). The role of metallothionein, glutathione, glutathione-S-transferases and DNA repair in resistance to platinum drugs in a series of L1210 lines made resistant to anticancer platinum agents. *Biochem. Pharmacol.*, **45**, 253–256.

KELLAND, L.R., BURGESS, L. & STEEL, G.G. (1987). Characterization of four new cell lines derived from human squamous carcinomas of the uterine cervix. *Cancer Res.*, **47**, 4947–4952.

KELLAND, L.R. & TONKIN, K.S. (1989). *In vitro* chemosensitivity of four new carcinoma of the cervix cell lines: relationship to radiosensitivity. *Eur. J. Cancer Clin. Oncol.*, **25**, 1211–1218.

KELLAND, L.R., JONES, M., ABEL, G. & HARRAP, K.R. (1992*a*). Human ovarian carcinoma cell lines and companion xenografts: a disease orientated approach to new platinum anticancer drug development. *Cancer Chemother. Pharmacol.*, **30**, 43–50.

KELLAND, L.R., MISTRY, P., ABEL, G., FRIEDLOS, F., LOH, S.Y., ROBERTS, J.J. & HARRAP, K.R. (1992*b*). Establishment and characterization of an *in vitro* model of acquired resistance to cisplatin in a human testicular non-seminomatous germ cell line. *Cancer Res.*, **52**, 1710–1716.

KELLAND, L.R., MISTRY, P., ABEL, G., LOH, S.Y., O'NEILL, C.F., MURRER, B.A. & HARRAP, K.R. (1992*c*). Mechanism-related circumvention of acquired *cis*-diamminedichloroplatinum(II) resistance using two pairs of human ovarian carcinoma cell lines by ammine/amine platinum (IV) dicarboxylates. *Cancer Res.*, **52**, 3857–3864.

KELLAND, L.R., MURRER, B.A., ABEL, G., GIANDOMENICO, C.M., MISTRY, P. & HARRAP, K.R. (1992*d*). Ammine/amine platinum (IV) dicarboxylates: a novel class of platinum complex exhibiting selective cytotoxicity to intrinsically cisplatin-resistant human ovarian cell lines. *Cancer Res.*, **52**, 822–828.

KELLEY, S.L., BASU, A., TEICHER, B.A., HACKER, M.P., HAMER, D.H. & LAZO, J.S. (1988). Overexpression of metallothionein confers resistance to anticancer drugs. *Science*, **241**, 1813–1815.

KELLEY, S.L. & ROZENCWEIG, M. (1989). Resistance to platinum compounds: mechanisms and beyond. *Eur. J. Cancer Clin. Oncol.*, **25**, 1135–1140.

KIKUCHI, Y., IWANO, I., MIYAUCHI, M., KITA, T., SUGITA, M., TENJON, Y. & NAGATA, I. (1990). Possible mechanisms of resistance to *cis*-Diamminedichloroplatinum (II) of human ovarian cancer cells. *Jpn. J. Cancer Res.*, **81**, 701–706.

KIRBY, K.S. & COOK, E.A. (1967). Isolation of deoxyribonucleic acid from mammalian tissues. *Biochem. J.*, **104**, 254–257.

KUPPEN, P.J.K., SCHUITEMAKER, H., VEER, L.J., BRUIJN, E.A., OOSTEROM, A.T. & SCHRIER, P.I. (1988). *Cis*-Diamminedichloroplatinum(II)-resistant sublines derived from two human ovarian tumor cell lines. *Cancer Res.*, **48**, 3355–3359.

LEWIS, A.D., HAYES, J.D. & WOLF, C.R. (1988). Glutathione and glutathione-dependent enzymes in ovarian adenocarcinoma cell lines derived from a patient before and after the onset of drug resistance: intrinsic differences and cell cycle effects. *Carcinogenesis*, **9**, 1283–1287.

LOH, S.Y., MISTRY, P., KELLAND, L.R., ABEL, G. & HARRAP, K.R. (1992). Reduced drug accumulation as a major mechanism of acquired resistance to cisplatin in a human ovarian carcinoma cell line: circumvention studies using novel platinum (II) and (IV) ammine/amine complexes. *Br. J. Cancer*, **66**, 1109–1115.

LOWRY, O.H., ROSEBROUGH, M.T., FARR, A.L. & RANDALL, R.J. (1951). Protein measurements with the folin phenol reagent. *J. Biol. Chem.*, **193**, 265–269.

MANGIONI, C., BOLIS, G., PECORELLI, S., BRAGMAN, K., EPIS, A., FAVALLI, G., GAMBINO, A., LANDONI, F., PRESTI, M., TORRI, W., VASSENA, L., ZANABONI, F. & MARSONI, S. (1989). Randomised trial in ovarian cancer comparing cisplatin and carboplatin. *J. Natl Cancer Inst.*, **81**, 1464–1468.

MASUDA, H., OZOLS, R.F., LAI, G.-M., FOJO, A., ROTHENBERG, M. & HAMILTON, T.C. (1988). Increased DNA repair as a mechanism of acquired resistance to *cis*-diamminedichloroplatinum(II) in human ovarian cancer cell lines. *Cancer Res.*, **48**, 5713–5716.

MASUDA, H., TANAKA, T., MATSUDA, H. & KUSABA, I. (1990). Increased removal of DNA-bound platinum in a human ovarian cancer cell line resistant to *cis*-diamminedichloroplatinum(II). *Cancer Res.*, **50**, 1863–1866.

MCGUIRE, W.P. III., ARSENEAU, J., BLESSING, J.A., DISAIA, P.J., HATCH, K.D., GIVEN, F.T. Jr., TENG, N.N.H. & CREASMAN, W.T. (1989). A randomized comparative trial of carboplatin and iproplatin in advanced squamous carcinoma of the uterine cervix: A Gynecologic Oncology Group Study. *J. Clin. Oncol.*, **7**, 1462–1468.

MISTRY, P., KELLAND, L.R., ABEL, G., SIDHAR, S. & HARRAP, K.R. (1991). The relationships between glutathione, glutathione-S-transferase and cytotoxicity of platinum drugs and melphalan in eight human ovarian carcinoma cell lines. *Br. J. Cancer*, **64**, 215–220.

PINTO, A.L. & LIPPARD, S.J. (1985). Binding of the antitumor drug *cis*-diamminedichloroplatinum(II) (cisplatin) to DNA. *Biochim. Biophys. Acta.*, **780**, 167–180.

ROBERTS, J.J., KNOX, R.J., FRIEDLOS, F. & LYDALL, D.A. (1986). DNA as the target for the cytotoxic and anti-tumour action of platinum co-ordination complexes: comparative *in vitro* and *in vivo* studies of cisplatin and carboplatin. In *Biochemical Mechanisms of Platinum Antitumour Drugs*, McBrien, D.C.H. & Slater, T.F. (eds), pp. 29–64. IRL Press: Oxford.

RUSSELL, J.M., BLAIR, V. & HUNTER, R.D. (1987). Cervical carcinoma: prognosis in younger patients. *Br. Med. J.*, **295**, 300–305.

SKEHAN, P., STORENG, R., SCUDIERO, D., MONKS, A., MCMAHON, J., VISTICA, D., WARREN, J.T., BOKESCH, H., KENNEY, S. & BOYD, M.R. (1990). New colorimetric cytotoxicity assay for anticancer drug screening. *J. Natl Cancer Inst.*, **13**, 1107–1118.

SMALES, E., PERRY, C.M., ASHBY, M.A. & BAKER, J.W. (1987). The influence of age on prognosis in carcinomas of the cervix. *Br. J. Obstet. Gynaecol.*, **94**, 784–787.

TEICHER, B.A., HOLDEN, S.A., KELLEY, M.J., SHEA, T.C., CUCCHI, C.A., ROSOWSKY, A., HENNER, W.D. & FREI, E. III (1987). Characterization of a human squamous carcinoma cell line resistant to *cis*-Diamminedichloro platinum(II). *Cancer Res.*, **47**, 388–393.

TEICHER, B.A., HOLDEN, S.A., HERMAN, T.S., ALVAREZ SOTOMAYOR, E., KHANDEKAR, V., ROSBE, K.W., BRANN, T.W., KORBUT, T.T. & FREI, E. III (1991). Characteristics of five human tumor cell lines and sublines resistant to *cis*-diamminedichloroplatinum(II). *Int. J. Cancer*, **47**, 252–260.

WELANDER, C.E. & PARKER, R.L. Jr. (1987). Human tumor clonogenic assay studies of cervical cancer. In *Cervix Cancer*, Surwit, E.A. & Alberts, D.S. (eds), pp. 185–197. Martinus Nijhoff: Boston.